# Clinical implications of repeated drug monitoring of imatinib in patients with metastatic gastrointestinal stromal tumour

**DOI:** 10.1186/s13569-016-0062-2

**Published:** 2016-12-15

**Authors:** Ivar Hompland, Øyvind Sverre Bruland, Kumari Ubhayasekhera, Jonas Bergquist, Kjetil Boye

**Affiliations:** 1 Department of Oncology, Norwegian Radium Hospital, Oslo University Hospital, PO Box 4953, Nydalen, 0424 Oslo, Norway; 2 Institute of Clinical Medicine, University of Oslo, Oslo, Norway; 3 Department of Chemistry, Biomedical Center, Analytical Chemistry and Science for Life Laboratory, Uppsala University, Uppsala, Sweden; 4 Department of Tumor Biology, Norwegian Radium Hospital, Oslo University Hospital, Oslo, Norway

**Keywords:** Gastrointestinal stromal tumour, Drug monitoring, Imatinib, Plasma concentration

## Abstract

**Background:**

Imatinib mesylate (IM) is the preferred treatment for the majority of patients with metastatic gastrointestinal stromal tumour (GIST). Low trough IM concentration (C_min_) values have been associated with poor clinical outcomes in GIST patients. However, there are few studies of repeated measurements of IM levels, and therapeutic drug monitoring is not yet a part of routine clinical practice. This study was conducted to reveal clinical scenarios where plasma concentration measurement of IM trough level (C_min_) is advantageous.

**Methods:**

Patients with advanced GIST receiving IM were included from January 2011 to April 2015. Heparin plasma was collected at each follow-up visit. Ninety-six samples from 24 patients were selected for IM concentration measurement. Associations between IM plasma concentration and clinical variables were analyzed by Students’ t test, univariate and multivariate linear regression analyses.

**Results:**

The mean IM C_min_ plasma concentrations for patients taking <400, 400 and >400 mg daily were 782, 1132 and 1665 ng/mL, respectively (p = 0.010). High IM C_min_ levels were correlated with age, low body surface area, low haemoglobin concentration, low creatinine clearance, absence of liver metastasis and no prior gastric resection in univariate analysis. In multivariate analysis age, gastric resection and liver metastasis were included in the final model. Eight patients had disease progression during the study, and mean IM levels were significantly lower at time of progression compared to the previous measurement for the same patients (770 and 1223 ng/mL, respectively; p = 0.020).

**Conclusions:**

Our results do not support repeated monitoring of IM levels on a routine basis in all patients. However, we have revealed clinical scenarios where drug measurement could be beneficial, such as for patients who have undergone gastric resection, suspicion of non-compliance, subjectively reported side effects, in elderly patients and at the time of disease progression.

## Background

Since the introduction of imatinib mesylate (IM) [1], the outcome of metastatic gastrointestinal stromal tumour (GIST) has improved considerably [2]. IM is an inhibitor of receptor tyrosine kinases, including the stem cell factor receptor *KIT* and the platelet-derived growth factor receptor alpha (*PDGFRA*), the main drivers of tumour development in GIST [3]. Several clinical trials have demonstrated the efficacy and safety of IM, and it has become the treatment of choice for the majority of patients with metastatic GIST [2, 4, 5]. The median duration of response to IM in metastatic GIST is 29 months [2], with approximately 20% of the responses lasting 10 years or more [6]. Still, most patients eventually progress on IM, requiring second- and third-line therapy with other tyrosine kinase inhibitors such as sunitinib and regorafinib [7].

In patients with chronic myeloid leukaemia (CML) and GIST, pharmacokinetic (PK) studies have shown that IM has >90% bioavailability following oral administration [8]. IM plasma concentration is influenced by various factors such age, body weight, body surface area (BSA), previous major gastric resection, white blood cell (WBC) count, haemoglobin, creatinine clearance, albumin, and alpha glycoprotein (AGP) levels [9–15]. A retrospective sub-study from the B2222 trial [4], the first trial showing safety and efficacy of IM in metastatic GIST patients, presented a significantly shorter time to progression in patients with IM trough levels (C_min_) below 1110 ng/mL at day 29 [16]. Additionally, a retrospective study in patients with CML in chronic phase reported that C_min_ of IM could predict clinical outcome [13]. However, the optimal threshold value of IM C_min_ has yet to be determined; both in patients with GIST and CML. A prospective PK study showed a significant decrease of approximately 30% in plasma IM concentration after 90 days of treatment [17], indicating that drug monitoring should preferentially be done after 3 months. This finding was recently supported by a study in real-life practice, where C_min_ was analysed after more than 3 months of treatment, and concentrations above 760 ng/mL were associated with longer progression-free survival (PFS) [18].

Although considerable inter-patient variability in IM plasma concentrations (40–60%) has been observed in several studies [15, 16], a fixed dose of 400 mg IM is the standard of care in patients with metastatic GIST [7]. Patients that progress on 400 mg/day and patients with KIT exon 9 mutations may benefit from increasing the dose to 800 mg/day [2, 19, 20]. Treatment with 400 mg IM is generally well tolerated, but patients still experience side effects such as anaemia, periorbital oedema, muscle cramps, and diarrhoea [2, 4, 5]. Several of these can be ameliorated with supportive measures, but some patients need dose modifications [21]. Compliance, i.e. adherence to self-administered drugs, is a general challenge for patients on any long-term treatment, as also reported for patients with GIST [22]. However, the extent of non-compliance is often not known and might be a larger problem than expected. Altogether, there are several situations where IM plasma concentration measurements might have a considerable clinical impact in patients with metastatic GIST. However, at present, therapeutic drug monitoring (TDM) is not yet a part of routine clinical practice.

The aim of this study was to assess IM plasma concentration repeatedly over several years in a group of patients with metastatic GIST and thereby revealing scenarios where such measurements might have clinical implications.

## Patients and methods

### Patients

Patients with GIST treated with IM were included from January 2011 to April 2015. Inclusion criteria were as follows: (1) histologically confirmed GIST; (2) treatment with IM initiated >90 days prior to study entry; (3) high-risk tumour in the need of adjuvant IM, metastatic disease or inoperable primary tumour. Fifty-three patients were enrolled, of whom 19 received IM in a neoadjuvant/adjuvant setting and 34 received IM for metastatic disease or inoperable primary tumour. For the present investigation we focused on patients in advanced or metastatic setting. We further excluded eight patients who had less than three available plasma samples and two patients where drug intake was not registered. Twenty-four patients were included in the final cohort. All patients attended regular 3- to 6-month follow-up visits and were seen by the same physician (ØSB). Radiological evaluation with computed tomography of the abdomen and pelvis was performed every 3–6 months depending on the clinical scenario. Disease progression was objectively documented by an experienced radiologist. Secondary review using RECIST or CHOI criteria was not performed. Clinicopathological data were collected retrospectively by reviewing medical records. Body weight, height and biochemical parameters were measured at the time of blood sampling for PK assessment. Creatinine clearance was estimated using the Cockcroft-Gault formula: estimated creatinine clearance = (140 − age in years) × (weight in kilograms) × (0.85 if female)/(72 − serum creatinine) [23]. The study was approved by the Regional Ethics Committee (#S-06133a), and written informed consent was obtained from all patients. Patients were asked if they took the drug as prescribed, and divided into three groups based on drug compliance: Excellent compliance: Never forget to take IM; Intermediate compliance: Forget to take IM on occasions, less than once a week; Poor compliance: Not taking IM regularly with gaps for several days.

### Sample collection

Three milliliter heparin plasma was collected at each follow-up visit. Within 1 h of the collection, the blood samples were centrifuged in room temperature for 15 min at 2500×*g*, and were stored at −20 °C until analysis. Samples were drawn in a routine clinical setting and not at the time of trough level. The time of drug intake was registered, and the validated Bayesian method developed by Gotta and colleagues [24] was used to extrapolate the measured concentrations to C_min_.

### Measurements of IM concentrations

The determination of the IM plasma concentrations followed the protocol as described in Ubhayasekhera et al. [25]. IM standard was kindly provided by Novartis (Basel, Switzerland). All chemicals including internal standard (Trazodone) and ultrapure solvents were purchased from Sigma Aldrich (Stockholm, Sweden), unless otherwise stated. The stock solutions of IM and internal standard were prepared by dissolving methanol to obtain a final concentration of 1 mg/mL. Protein precipitation was applied as a sample pretreatment. Twenty-five microliter of methanol containing 1 µg/mL internal standard and 0.5 mL of methanol were added to 100 µL of plasma, shaken in 10 min and centrifuged for 10 min at 4 °C at 14,000*g*. The supernatant was dried by vacuum centrifugation and the residue was reconstituted in 100 µL of 5% acetonitrile containing 0.1% formic acid. Aliquouts of 10 µL were injected into the LC–MS system. Chromatography and mass spectrometry was performed as previously described [25, 26].

### Statistical analysis

All statistical analyses were performed by using SPSS 21.0 (SPSS, Chicago, IL, USA). Differences in plasma concentrations between dose groups were assessed by Kruskal–Wallis test. The IM C_min_ values were log-transformed for the subsequent analyses. To assess the characteristics of the plasma samples in a homogenous cohort, we focused on the samples being drawn in patients taking 400 mg daily (n = 69). Correlations between IM C_min_ and other variables were analysed by univariate linear regression (Pearson) and independent samples Student’s t test. Variables that showed significant correlations (p < 0.05) with IM C_min_ in univariate analysis were included in a multivariate analysis using a multiple linear regression model with stepwise, backward elimination of variables. Correlations were also tested using a more stringent linear mixed models effect analysis to take into account intra-patient correlation. All tests were two-sided, and p values less than 0.05 were considered statistically significant.

## Results

### Patient characteristics

Ninety-six samples from 24 patients included in the study were analysed. There were 4 patients with three samples, 16 patients with four samples and 4 patients with five samples. The median duration of IM treatment prior to the first sample was 25 months (range 3–77 months). The median time from the first sample to the last sample was 32 months (range 4–48 months). All patients received IM for metastatic disease, except one patient who was medically inoperable and received IM for a large GIST in the small bowel. The median age was 69 years (range 33–88). The clinical and pathological features of all patients are listed in Table 1. Sixteen patients reported excellent compliance, seven had intermediate compliance and one patient poor compliance. No patients experienced serious life-threatening adverse events. Seven patients had dose reductions: Six patients from 400 to 200 mg due to self-reported side effects and one patient from 800 to 400 mg due to severe fluid retention and haematological toxicity.

**Table 1:** Baseline clinical and pathological characteristics of the 24 patients enrolled in the study

Characteristic	Number (%)
Gender	
Female	8 (33)
Male	16 (67)
Primary tumour site	
Stomach	8 (33)
Small bowel	13 (54)
Rectum	2 (8)
Unknown	1 (4)
Histological subtype	
Spindle cell	17 (71)
Epitheloid	1 (4)
Mixed	3 (13)
ND	3
Mutation analysis	
KIT exon 11	18 (75)
KIT exon 9	2 (8)
PDGFRA exon 12	1 (4)
Mutations not detected	2 (8)
ND	1
Metastatic site	
Liver	13 (54)
Intraperitoneal cavity	7 (29)
Liver + intraperitoneal cavity	3 (13)
No metastasis (inoperable primary tumour)	1 (4)

*ND* not determined

### C_min_ plasma concentrations

**Fig. 1:**
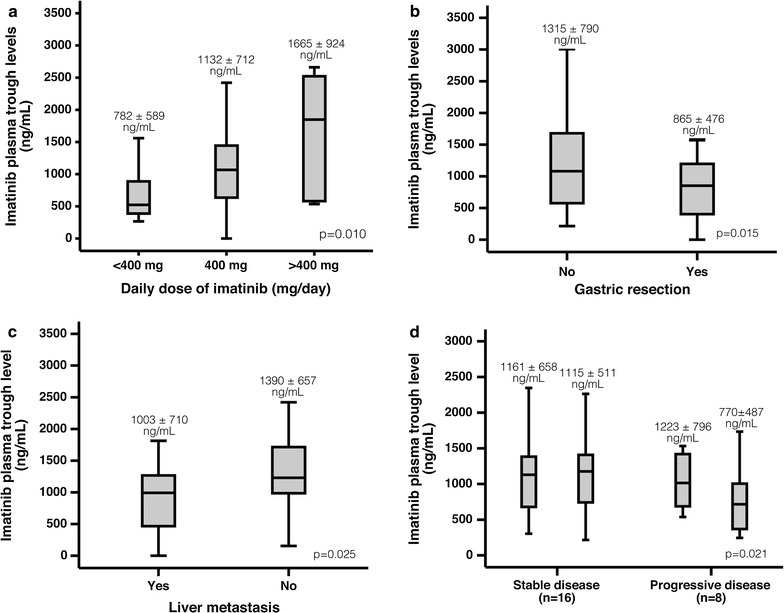
*Boxplots* showing imatinib mesylate (IM) trough levels (C_min_). *Boxes* indicate the median, the 25th and 75th percentile, and *whiskers* represent maximum and minimum values. Outliers are censored. Mean C_min_ values ± standard deviations are indicated for each category. **a** IM C_min_ levels categorised according to dose groups: <400 mg (n = 21), 400 mg (n = 69) and >400 mg (n = 6). **b** IM C_min_ levels categorised according to gastric resection (n = 28) or not (n = 41). **c** IM C_min_ levels categorised according to presence (n = 46) or absence (n = 23) of liver metastases. **d** IM C_min_ levels categorised according to whether patients had experienced disease progression or not. For patients with disease progression (*right panel*), the *left box* represents the last plasma samples at stable disease and the *box to the right* represents the plasma samples at the time of progressive disease. For patients with stable disease (*left panel*), the *left box* represents the second last plasma samples and the *box to the right* represent the last plasma samples drawn

Plasma samples were grouped according to the IM dose at time of sampling: <400 mg group (100 mg: n = 2, 200 mg: n = 19), 400 mg (n = 69) and >400 mg (600 mg: n = 1, 700 mg: n = 1 and 800 mg: n = 4). Mean ± standard deviation values of IM C_min_ plasma concentrations were 782 ± 589, 1132 ± 712 and 1665 ± 924 ng/mL, respectively (Fig. 1a). The difference between the groups was statistically significant (p = 0.010). Intra-patient and inter-patient variability was relatively large. The mean intra-patient variability (coefficient of variation) in patients taking 400 mg was 36% and the highest intra-patient variability 69%, with maximum plasma concentration 1188 ng/mL and minimum of 195 ng/mL. The mean inter-patient variability in patients taking 400 mg was 68%, with the highest measured concentration of 4491 ng/mL and the lowest concentration 195 ng/mL. Among the six patients with a dose reduction to 200 mg, two had relatively high mean plasma levels of 1418 and 2242 ng/mL, whereas the other four had mean plasma concentrations of 387, 437, 565 and 521 ng/mL. Two patients started on 200 mg and had mean plasma concentrations of 1704 and 540 ng/mL.

### Patient characteristics and C_min_ plasma concentrations

Correlations between IM C_min_ and clinical characteristics were analysed in patients receiving the standard dose 400 mg. The results presented below refer to the per-sample analysis. Linear mixed model effects analyses gave similar trends, although without reaching statistical significance. In univariate analysis, high IM C_min_ was significantly correlated with age (β = 0.303, p = 0.012), BSA (β = −0.300, p = 0.010), low haemoglobin concentration (β = −0.290, p = 0.016), low creatinine clearance (β = −0.234, p = 0.050), but not with albumin (p = 0.061) or calcium level (p = 0.999), tumour diameter (p = 0.368), gender (p = 0.915), WBC (p = 0.832) or platelet count (p = 0.816).

Nine patients (38%) had undergone subtotal or total gastrectomy, and IM C_min_ was significantly lower in these patients (865 ± 476 ng/mL; n = 28) than in plasma samples from patients without gastric surgery (1315 ± 790 ng/mL; n = 41; p = 0.015) (Fig. 1b). Furthermore, IM C_min_ was significantly lower in the plasma samples from patients with liver metastases (1003 ± 710 ng/mL, n = 46) compared to patients without liver metastases (1390 ± 657 ng/mL, n = 23; p = 0.025) (Fig. 1c).

Multivariate analysis was performed including variables that were associated with IM C_min_ in univariate analysis in the 400 mg group. Gastric resection (p = 0.021), age (p = 0.049) and liver metastases (p = 0.010) were the covariates significantly associated with IM C_min_.

### Disease progression and C_min_ plasma concentrations

Eight patients had disease progression during the study. In seven of these IM C_min_ concentrations decreased at the time of progression compared to the previous measurement. The mean IM C_min_ concentration at the time of progression was 770 ± 487 ng/mL, and in the last sample from the time of stable disease from the same patients 1223 ± 796 ng/mL (p = 0.021; Student’s test). In comparison, there was no statistically significant difference in IM C_min_ concentration between the two last plasma samples collected in patients with stable disease throughout the study (1161 ± 658 versus 1115 ± 511 ng/mL) (Fig. 1d).

## Discussion

The role of IM C_min_ measurements in optimizing therapeutic efficacy in GIST is still investigational, despite preliminary estimates of IM blood levels that are associated with improved clinical outcomes (C_min_ > 1110 ng/mL) [16]. In this study, we assessed C_min_ in a group of patients over several years trying to determine whether there are clinical scenarios where measurements of IM C_min_ could be advantageous. To the best of our knowledge this is the first study in metastatic GIST with repeated measurements of C_min_ plasma concentrations including samples at the time of documented progression.

Low IM C_min_ was associated with major gastrectomy in both univariate and multivariate analysis, which is consistent with previous findings [14]. IM tablets dissolve more rapidly at pH 5.5 or less [8], and lack of gastric acid secretion may explain the lower concentration in such patients. Many patients with metastatic GIST have previously undergone surgery for a primary gastric GIST and one might speculate that such patients could possess an increased risk of sub-therapeutic IM plasma levels and subsequently a less favourable disease outcome. In our cohort, only eight patients had disease progression, of which three had undergone gastric resection. Thus, analysing the prognostic impact of gastric surgery is impossible due to small patient numbers. Still, a more individualized drug dosage based on IM plasma concentrations may be beneficial in patients with prior gastric surgery.

Interestingly, in the current study patients with liver metastases had low IM C_min_ compared to patients without liver metastases. A previous study has shown that IM clearance is not affected by low volume liver disease [17], and it thus seems unlikely that the liver metastases per se affect IM metabolism in our patients. We are not aware of studies that have reported differences in IM plasma concentration in patients with or without liver metastases, and this issue could be of interest for further studies.

Older patients had higher plasma concentrations in our cohort. The well-known decline of organ functions and increased prevalence of comorbidity and concomitant medication among elderly patients may influence the pharmacokinetics and pharmacodynamics of IM. We did not prospectively register concomitant medications or co-occurring medical conditions, and are thus not able to discern the role of these factors separately. Our results could suggest that individual dosing supported by IM plasma concentration measurement could be even more useful in elderly patients, to balance side effects and anti-tumour efficacy more precisely.

Although there were no serious adverse events in our study, dose reduction due to subjective side effects were mandatory in seven patients. Two of these patients had relatively high C_min_ on 200 mg, suggesting that for some patients this dose is enough to ensure optimal therapeutic plasma levels of IM. The four other patients had low levels suggesting that patient-reported side effects are not necessarily associated with high plasma concentrations. Few studies have explored the relationship between IM plasma concentration and side effects. One study showed that the occurrence and number of side effects correlated with IM total and free plasma concentrations in GIST patients [27], but further studies on relations between concentration and toxicity are warranted. Unfortunately, we did not register side effects in a formal and prospective manner. However, measuring C_min_ concentrations in patients experiencing subjective side effects (e.g. muscle cramps, dizziness, fatigue etc.) that limit daily activity may help to determine whether it is safe to modify the dose of IM.

The relatively large inter- and interpatient variability in our study compared to other real-life cohorts [14, 18] could be explained by lack of compliance. Although oral cancer therapies offer patients the convenience of self-administration at home, evidence show that adherence to these therapies is far from optimal [28, 29]. The BFR14 Study evaluated the effect of IM interruption in responding patients (complete response, partial response, or stable disease) after different periods of treatment (1, 3 and 5 years), and results from the study indicated that discontinuation of IM is associated with rapid progression [30–32]. Therefore, maintaining proper adherence may be of great significance and drug monitoring could potentially improve compliance to therapy.

Interestingly, we found a decrease in C_min_ plasma concentration at the time of disease progression, which might explain loss of disease control in certain patients. Measurements of IM C_min_ in case of progressive disease could therefore be indicated if lack of patient compliance has been ascertained. A sub-therapeutic drug level at the prescribed dose could suggest that increasing the dose would be of clinical benefit, in particular in the absence of secondary KIT or PDGFRA mutations. Studies comparing 400 with 800 mg IM daily in advanced disease showed no clinical benefit of IM 800 mg daily, except for tumours with KIT exon 9 mutations [33]. Despite this, dose escalation to 800 mg can be beneficial in up to 30% of patients upon disease progression on 400 mg [2, 19, 20]. IM plasma concentration measurements have not been performed in dose escalation studies, and perhaps only patients with sub-therapeutic IM levels will benefit from this strategy, whereas the remainder should be offered second-line therapy.

Total IM plasma concentration was measured in our study. Another option is to measure free drug concentrations; i.e. the pharmacologically active fraction not bound by albumin or AGP. The area under the PK curve (AUC) for IM, which can either be measured directly or as the correction of the total drug concentration for binding to AGP, may provide a better surrogate for cellular drug exposure than total IM concentration [15, 26]. Furthermore, IM concentration measurement in the cytoplasm of the tumour cells could even more precisely predict target inhibition and clinical efficacy. A new approach to measurement of intracellular levels of IM in an in vivo setting has been developed, and there were large variation in IM concentrations between plasma, adipose tissue, and different sites within a given tumour [34]. Although only three patients were included in the latter study, this highlights the importance of further clinical investigations on measurements of intracellular IM levels in GIST tissues to understand their possible impact on patient outcome.

Among the limitations of this study are the retrospective registration of the majority of the clinical data and side effects. We neither did review of the radiology nor the pathology, but experienced sarcoma radiologists and pathologists at a major reference centre had already confirmed the diagnostic work-up at start of IM, including analyses of KIT and PDGFRA mutations that were found in all patients except three. Furthermore, patients with <3 plasma samples were excluded, and median treatment duration of IM before inclusion was 25 months. Even though patients were not excluded due to progressive disease, a bias towards patients without progression could have occurred. Moreover, the plasma samples were not drawn at trough time, but a validated method to extrapolate the samples to trough was used [25]. Even though these issues in general would be considered as shortcomings, they reflect well a routine oncology practice, and our findings could therefore easily be transferred to a routine clinical setting.

In conclusion, our results do not support repeated monitoring of IM levels on a routine basis in all patients. However, we have revealed clinical scenarios where drug measurement could be beneficial, such as for patients who have undergone gastric resection, suspicion of non-compliance, subjectively reported side effects, in elderly patients and at the time of disease progression. Whether dose escalation could be beneficial at disease progression for patients with low IM plasma concentration should be further studied.

## Abbreviations

GIST: gastrointestinal stromal tumour
IM: imatinib mesylate
C_min_: trough level
PDGFRA: platelet-derived growth factor receptor alpha
CML: chronic myeloid leukaemia
PK: pharmacokinetic
BSA: body surface area
WBC: white blood cell
AGP: alpha glycoprotein
PFS: progression-free survival
TDM: therapeutic drug monitoring
